# P-533. Inpatient Burden of Respiratory Syncytial Virus, COVID-19, or Influenza in the United States Among Children < 5 Years of Age

**DOI:** 10.1093/ofid/ofaf695.748

**Published:** 2026-01-11

**Authors:** Kathleen M Andersen, Maria D McColgan, Maya Reimbaeva, Tara Ahi, Mary M Moran, Alejandro D Cane, Santiago M C Lopez

**Affiliations:** Pfizer Inc., New York, NY; Pfizer Inc., New York, NY; Pfizer, Inc., Groton, Connecticut; Pfizer Inc, New York, NY; Pfizer Inc., New York, NY; Pfizer, Collegeville, Pennsylvania; Pfizer Inc, New York, NY

## Abstract

**Background:**

Respiratory syncytial virus (RSV), COVID-19 and influenza are leading causes of acute respiratory illness in children. COVID-19 has been shown to have greater inpatient burden than influenza among children age < 5 years, however the comparative burden with RSV is unknown.

**Figure:**
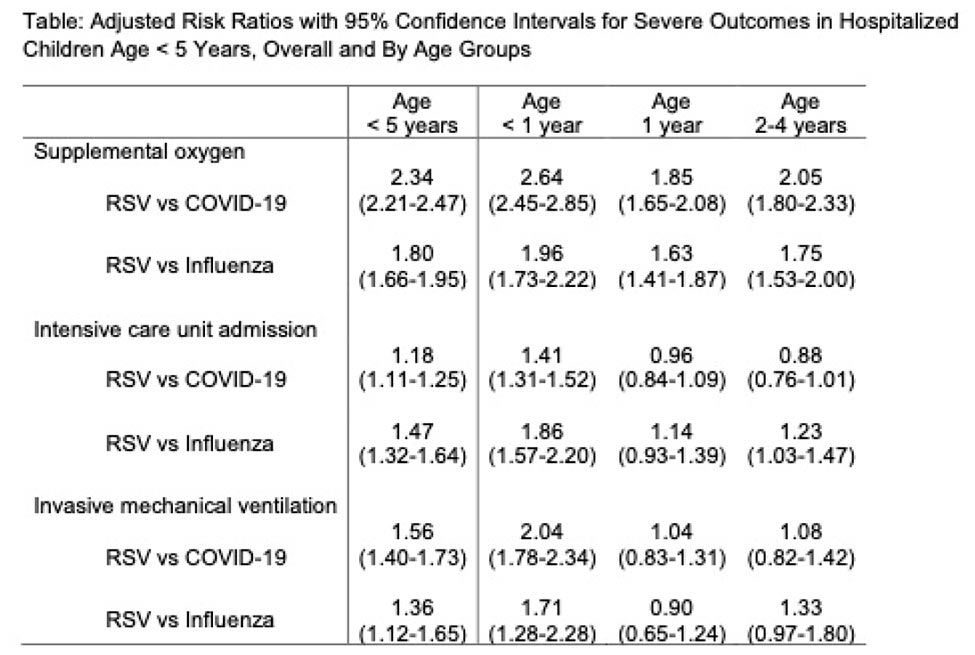

**Methods:**

We defined a retrospective cohort of children age < 5 years hospitalized for RSV or influenza (April 2019 – March 2020, given disruptions in circulation during the COVID-19 pandemic), or COVID-19 (April 2021 – July 2023). We used PINC-AI Healthcare Database, which contains deidentified hospital records covering ∼25% of admissions in the United States. Outcomes of interest were length of stay (LOS), supplemental oxygen use, intensive care unit (ICU) admission, invasive mechanical ventilation (IMV) and in-hospital mortality. Adjusted risk ratios (aRR) with 95% confidence intervals (CI) were estimated using weighted robust Poisson regression to compare RSV to COVID-19 and RSV to influenza.

**Results:**

We identified 33,644 hospitalized children (19,015 RSV; 10,316 COVID-19; 4,313 influenza). In weighted models to reduce potential confounding, risk of supplemental oxygen, ICU admission and IMV remained higher with RSV than either COVID-19 or influenza in children < 5 years. As compared with COVID-19, RSV was associated with > 2-fold increased risk of supplemental oxygen use (aRR 2.34, 95% CI 2.21-2.47) and a more than 1.5-fold higher risk of IMV (aRR 1.56, 95% CI 1.40-1.73). Similarly, in comparison with influenza, patients hospitalized for RSV had a higher risk of all measures of healthcare resource utilization. In subgroup analyses (aged < 1; 1 and 2-4 years), risks remained elevated, particularly for children aged < 1 year, as compared to older age groups. In-hospital mortality with COVID-19 occurred for 0.5% of patients, compared to RSV (0.1%) or influenza (0.3%).

**Conclusion:**

Risks for in-hospital resource utilization were higher with RSV than either COVID-19 or influenza in adjusted analyses. In-hospital death was greater for COVID-19 than RSV or flu. The severe outcomes observed in this study underscore the need for preventive measures such as maternal and childhood vaccination as well as monoclonal antibodies.

**Disclosures:**

Kathleen M. Andersen, PhD, MSc, Pfizer Inc.: Employee of Pfizer Inc. and may hold stock or stock options Maria D. McColgan, MD, MSEd, Pfizer Inc.: All authors are employees of Pfizer Inc. and may hold stock and/or stock options of Pfizer Inc. Maya Reimbaeva, MS, Pfizer Inc.: All authors are employees of Pfizer Inc. and may hold stock and/or stock options of Pfizer Inc. Tara Ahi, MPH, Pfizer Inc.: Employee of Pfizer Inc. and may hold stock or stock options Mary M. Moran, MD, Pfizer Inc.: Employee of Pfizer Inc. and may hold stock or stock options Alejandro D. Cane, MD, PhD, Pfizer Inc.: All authors are employees of Pfizer Inc. and may hold stock and/or stock options of Pfizer Inc. Santiago M.C. Lopez, MD, Pfizer Inc.: Employee of Pfizer Inc. and may hold stock or stock options|Pfizer Inc.: Stocks/Bonds (Public Company)

